# Microphakia and congenital cataract formation in a novel Lim2^C51R^ mutant mouse

**Published:** 2011-05-04

**Authors:** Oliver Puk, Nafees Ahmad, Sibylle Wagner, Martin Hrabé de Angelis, Jochen Graw

**Affiliations:** 1 Helmholtz Center Munich, German Research Center for Environmental Health, Institutes of Developmental Genetics, Neuherberg, Germany; 2 Helmholtz Center Munich, German Research Center for Environmental Health, Institutes of Experimental Genetics, Neuherberg, Germany; 3 Chair of Experimental Genetics, Technical University Munich, Center of Life and Food Sciences, Freising-Weihenstephan, Germany

## Abstract

**Purpose:**

Within a mutagenesis screen, we identified the new mouse mutant *Aca47* with small lenses and reduced axial eye lengths. The aim of the actual study was the molecular and morphological characterization of the mouse mutant *Aca47*.

**Methods:**

We analyzed the offspring of paternally *N*-ethyl-*N*-nitrosourea (ENU) treated C57BL/6J mice for eye-size parameters by non-invasive in vivo laser interference biometry. Linkage analysis of the eye size mutant *Aca47* was performed using single nucleotide polymorphisms and microsatellite markers. The *Aca47* mutation was identified by sequence analysis of positional candidate genes. A general polymorphism at the mutated site was excluded by restriction analysis. Eyes of the *Aca47* mouse mutant were characterized by histology. Visual properties were examined in the virtual drum.

**Results:**

We identified a new mutant characterized by a significantly smaller lens and reduced axial eye length without any changes for cornea thickness, anterior chamber depth or aqueous humor size. The smaller size of lens was more pronounced in the homozygous mutants, which further developed congenital cataracts in the lens nucleus. The mutation was mapped to chromosome 7 between the markers *D7Mit247* and *D7Mit81*. Using a positional candidate approach, the lens intrinsic integral membrane protein MP19 encoding gene *Lim2* was sequenced; a T→C exchange at cDNA position 151 leads to a cysteine-to-arginine substitution at position 51 of the Lim2 protein. Eye histology of adult heterozygous mutants did not show alterations on the cellular level. However, homozygous lenses revealed irregularly arranged lens fiber layers in the cortex. Virtual vision tests indicated that visual properties are not affected by reduced eye size of heterozygous individuals.

**Conclusions:**

These findings demonstrate a novel missense mutation in the *Lim2* gene that affects lens development in a semidominant manner. Since homozygous mutants develop congenital lens opacities, this line can be used as a model for inherited cataract formation in humans.

## Introduction

The general estimates for blindness gives a total number of ~50 Mio people worldwide. Recent data from the World Health Organization (WHO) indicate that cataracts represent the most frequent reason for blindness worldwide (39%), followed by uncorrected refractive error (18% - excluding presbyopia), glaucoma (10%), and age-related macular dystrophy AMD (7%) [1]. In addition to the implications for health care delivery and health care costs, cataract has been shown to be associated with falls and increased mortality, possibly because of associated systemic conditions. While major risk factors for age-related cataracts are diabetes and UV light (for a review see [2]), most lens opacifications in newborns are genetically caused.

In the mouse, various models for congenital cataracts are well established. The majority of the underlying mutations affect genes coding for transcription factors, structural proteins, or membrane proteins (for a review see [3]). One of the most abundant components in lens fiber cell membranes is lens intrinsic membrane protein-2 (Lim2; variously referred to as MP17, MP18, MP19, or MP20 [4]; ), which is a member of the peripheral myelin-22 (Pmp22) claudin family of mammalian transmembrane proteins, also known as pfam00822 [5,6]. Members of this family share a predicted topology characterized by four transmembrane helices and two extracellular domains or loops. Little is known about the function of Lim2. Studies with bovine lenses indicated that this protein localizes to junctional regions of the lens fiber cell membrane as well as throughout fiber cell membranes, suggesting a role in lens junctional communication [7,8]. Biochemical investigations further revealed that Lim2 is a glycophosphoprotein [9], which binds galectin-3 [10,11] as well as calmodulin [12,13] and forms homo-oligomers at least at the size of hexamers [14].

Recently, a semidominant p.G15V missense mutation in the mouse gene for Lim2 (*Lim2*) was identified. The underlying mouse mutant (*To3*) was characterized by severe congenital cataracts and microphthalmia [15]. In contrast, mice lacking *Lim2* exhibited only faint, central pulverulent cataracts. Moreover, this knockout mutation was associated with a recessive mode of inheritance [16].

Here we demonstrate a novel semidominant missense mutation in the *Lim2* gene of the mouse. We identified the mutation in a breeding colony of mice after paternal ENU treatment. Heterozygous mutants are characterized by smaller lenses and reduced axial eye lengths. We further identified congenital cataracts in homozygous carriers. Therefore, we suggest that the new mutant line can be used as a well characterized model for inherited cataract formation.

## Methods

### Mice

Mice were kept under specific pathogen-free conditions at the Helmholtz Center Munich, Munich, Germany. The use of animals was in accordance with the German Law of Animal Protection, the ARVO Statement for the Use of Animals in Ophthalmic and Vision Research, and the tenets of the Declaration of Helsinki. Male C57BL/6J mice were treated with ENU (80 mg/kg bodyweight applied by intraperitoneal injection in three weekly intervals) at the age of 10–12 weeks as previously described [17] and mated to untreated female C57BL/6J mice [18]. The offspring of the ENU-treated mice were screened at the age of 11 weeks for abnormalities of the eye size.

### Eye size determination

The sizes of ocular parameters were examined using laser interference biometry (LIB; “ACMaster,” Meditec; Carl Zeiss, Jena, Germany). Briefly, mice were anesthetized with an intraperitoneal injection of 137 mg ketamine and 6.6 mg xylazine per kilogram bodyweight. The anesthetized mouse was placed on a platform and oriented in an appropriate position using light signals from six infrared LEDs arranged in a circle that must be placed in the center of the pupil. Central measurements of lens thickness (polar diameter), axial length, corneal thickness, and anterior chamber depth as well as data evaluation were performed essentially as described previously [19,20]. Mice with phenotypic deviations were tested for a dominant mode of inheritance.

### Linkage analysis

Heterozygous carriers (first generation) were mated to wild-type C3HeB/FeJ mice, and the offspring (second generation) were backcrossed to wild-type C3HeB/FeJ mice. DNA was prepared from tail tips of affected offspring of the third generation (G3). For linkage analysis, genotyping of a genome-wide mapping panel consisting of 153 single nucleotide polymorphisms (SNP) was performed using MassExtend, a MALDI-TOF (matrix-assisted laser/desorption ionization, time of flight analyzer) mass spectrometry high-throughput genotyping system supplied by Sequenom (San Diego, CA) [21]. Fine mapping was performed with the microsatellite markers *D7Mit81*, *D7Mit91*, *D7Mit229*, and *D7Mit247*.

### Genotyping and sequencing

Genomic DNA was isolated from tail tips of JF1, C57BL/6J, DBA/2J, CFW, and C3HeB/FeJ wild-type mice or homozygous/heterozygous mutants according to standard procedures. For sequencing of the cDNA of the positional candidate gene, *Lim2*, the primer pairs Lim2–1 (5′-GAA GGA GGG CTC AGA ACA GA-3′) and Lim2–2 (5′-TCT AGG TCC TCC CCT TCC TC-3′) were used. PCR was performed with a PTC-225 thermocycler (Biozym, Hessisch Oldendorf, Germany). Products were analyzed by electrophoresis on a 1.5% agarose gel. Sequencing was performed commercially (GATC Biotech, Konstanz, Germany) after direct purification of the PCR products (Nucleospin Extract II, Macherey-Nagel, Düren, Germany).

To confirm the mutation in the genomic DNA, a 373-bp fragment was amplified from genomic DNA using the primer pairs Aca47-Genot1 (5′-CCC AAC CTT CCT TTC ACT CA-3′) and Aca47-Genot2 (5′-AGC TAT CAT GCT TTC CCT GTG-3′) and digested by the restriction enzyme AfeI.

### Impact of the mutation on protein structure and functionality

To generally assess the probable effect of the amino acid substitution on Lim2 protein function, we determined the SIFT (Sorting Intolerant from Tolerant) score. SIFT aligns sequences homologous to the protein of interest from the databases and predicts whether a specific amino acid substitution will be tolerated by calculating normalized probabilities (range from 0 to 1) for each substitution at a particular position. Scores below a threshold of 0.05 are predicted to be deleterious [22,23]. For initial description of Lim2 secondary structure alterations, we used the DiANNA web server for disulfide connectivity prediction. For each pair of cysteine in the protein sequence, a neural network trained to recognize disulfide bonds produces a score ranging from 0 to 1. A high score indicates high prediction reliability of disulfide bond formation [24].

### Histological analysis

Eyes of ten-week-old mice were analyzed histologically for eye pathologies. Prepared eyes were fixed for seven days in Davidson solution and embedded in JB-4 plastic medium (Polyscience Inc. Eppelheim, Germany) according to the manufacturer’s protocol. Sectioning was performed with an ultramicrotome (OMU3; Reichert-Jung, Walldorf, Germany). Serial transverse 3-µm sections were cut with a glass knife and stained with methylene blue and basic fuchsin. The sections were evaluated with a light microscope (Axioplan; Carl Zeiss, Jena, Germany). Images were acquired by means of a scanning camera (AxioCam; Jenoptik, Jena, Germany) and imported into an image-processing program (Photoshop 10.0; Adobe, Unterschleissheim, Germany).

### Vision test

Vision tests were performed between 9 AM and 4 PM using a virtual optomotor system (Cerebral Mechanics, Lethbridge, Canada) as described previously [25]. Briefly, a rotating cylinder covered with a vertical sine wave grating was calculated and drawn in virtual three-dimensional space on four computer monitors facing to form a square. Visually unimpaired mice track the grating with reflexive head and neck movements (head-tracking). Vision threshold of the tested mice was quantified by a simple staircase test. Rotation speed and contrast were set to 12.0 d/s and 100%, respectively. Since no significant threshold differences were observed between males and females (p>0.05; *t*-test), data of both sexes were combined. Thresholds of wild-type C57BL/6J and heterozygous *Aca47* mice were compared using the *t*-test.

### General

Chemicals and enzymes were from Fermentas (St-Leon-Rot, Germany), Merck (Darmstadt, Germany), or Sigma Chemicals (Deisenhofen, Germany). Oligonucleotides were synthesized by Sigma Genosys (Steinheim, Germany).

## Results

**Figure 1:**
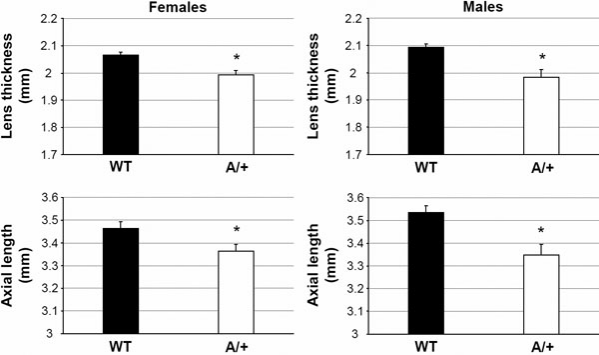
Axial eye length and lens thickness in *Aca47* mutants. Both parameters are given for wild-types (WT) and heterozygous mutants (A/+), both for females and males. Eye size determination of homozygous mutants was prevented by congenital cataracts. Female axes and lenses are slightly smaller than those of males. An asterisk (*) indicates statistical significance at p<0.001 (*t*-test).

We have screened offspring from ENU-treated male mice by LIB to detect dominant eye size anomalies. One of the confirmed mutants, *Aca47*, was characterized by clear, but significantly smaller lenses and reduced axial eye lengths (heterozygous mice; Figure 1). Eye size of homozygous *Aca47* individuals could not be determined due to irregular LIB signals caused by congenital cataract formation. Both heterozygous and homozygous mutants are fully fertile and viable.

**Figure 2:**
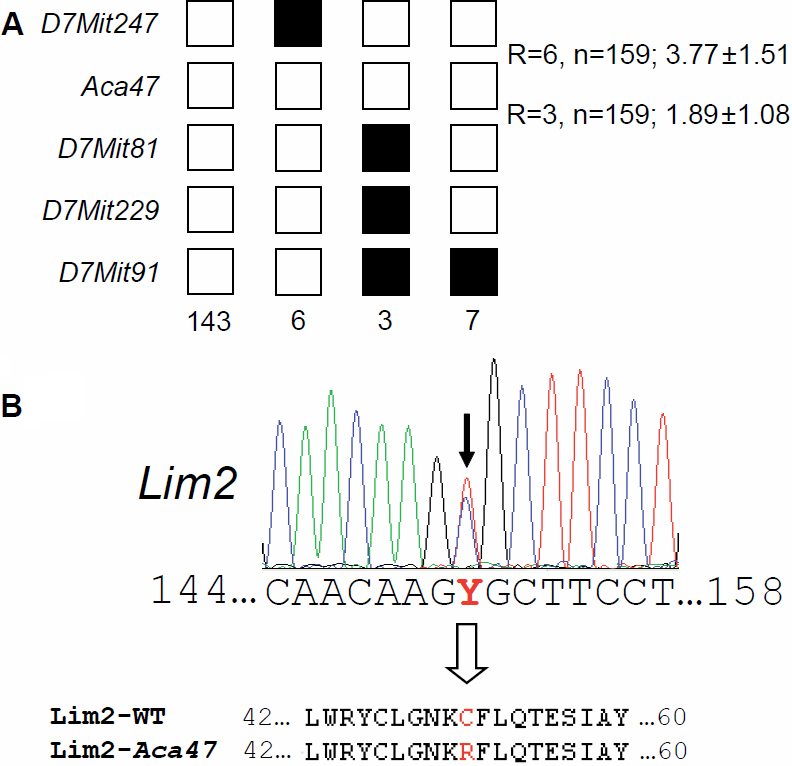
Fine mapping of the *Aca47* mutation and its effect on the *Lim2* gene. **A**: Haplotype analysis revealed a critical interval of 14.9 Mb between the markers *D7Mit247* and *D7Mit81* including the candidate gene *Lim2*. Black boxes illustrate the presence of two C3H marker alleles (recombination between microsatellite marker and *Aca47*); white boxes illustrate presence of one copy of both alleles, C3H and C57BL/6J (lack of recombination). The number of G3 progeny carrying the particular recombination pattern is given below the boxes. The total number of recombination events (R) between neighbored markers is shown on the right of the boxes, including the calculated relative genetic distances (cM). **B**: Sequence analysis of the *Lim2* coding region demonstrates a T->C exchange at cDNA position 151 (black arrow) resulting in a Cys to Arg amino acid exchange in the Lim2 protein (position 51; white arrow).

**Figure 3:**
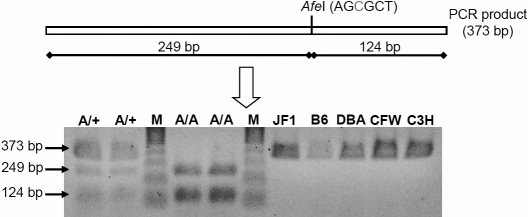
Exclusion of the T→C exchange as a polymorphism by restriction digest. In the mutants, the *Afe*I restriction enzyme cuts a genomic fragment of 373 bp that contains parts of *Lim2* exon 2 and its flanking regions; restriction digest results into two fragments of 249 bp and 124 bp. DNA restriction analysis in different wild type strains of mice showed the absence of a T151C exchange. However, it is present in two heterozygous (A/+) and two homozygous (A/A) *Aca47* mutant mice randomly collected from the actual running breeding. B6, C57BL/6J; DBA, DBA/2J; C3H, C3HeB/FeJ; M: Sigma Gene Ruler 100 bp DNA Ladder Plus marker.

In a genome-wide linkage analysis using SNP markers, the mutation was mapped to chromosome 7 close to the SNP marker rs13479256 (60.7 Mb, Build 37.1). Fine mapping with microsatellite markers revealed a critical interval of 14.9 Mb between *D7Mit247* (37.9 Mb) and *D7Mit81* (52.8 Mb; Figure 2A) making the lens intrinsic membrane protein 2 encoding gene *Lim2* a very interesting candidate gene. Sequencing of the *Lim2* cDNA identified a T→C exchange at position 151 (Figure 2B) that does not represent a general polymorphism, since it was not detected in wild-type mice of several strains (JF1, C57BL/6J, DBA, CFW, and C3HeB/FeJ; Figure 3). The substitution leads to a cysteine-to-arginine exchange at position 51 of the Lim2 amino acid sequence (p.C51R; Figure 2B). A SIFT impact analysis on general protein functionality yielded a score of 0.05 for p.C51R proving that the replacement of the polar (uncharged) cysteine residue by a polar (charged) arginine is not tolerable. p.C51R most likely prevents disulfide bridge formation, since computer-assisted secondary structure analysis determined an optimal disulfide bond reliability between Cys51 and Cys11 (DiANNA score=0.997) or between Cys51 and Cys46 (DiANNA score >0.999) in the wild-type Lim2 protein.

**Figure 4:**
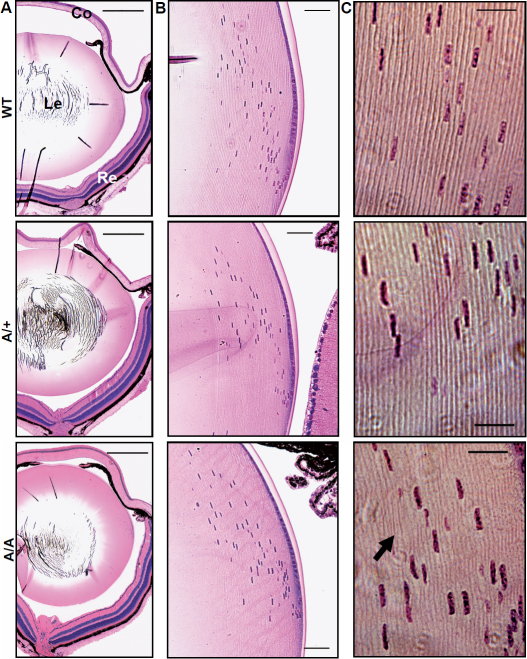
Histological analysis of *Aca47* eyes. Wild-type (WT), heterozygous (A/+), and homozygous mutant eyes are compared at ten weeks of age. **A**: The overview of the analyzed eye sections indicate that histology of cornea (Co) and retina (Re) is not altered in the mutants. Le: lens; bars=0.5 mm. **B**: Magnification of the lens bow regions reveal regular degradation patterns of lens fiber cell nuclei in the mutants. Bars=50 µm. **C**: Lens fiber layers at the equatorial outer cortex are regularly arranged in wild-types and heterozygous mutants. In homozygous individuals, fiber layers are partially thickened (arrow) and show less regular, wave-like structures. Bars=20 µm.

**Figure 5:**
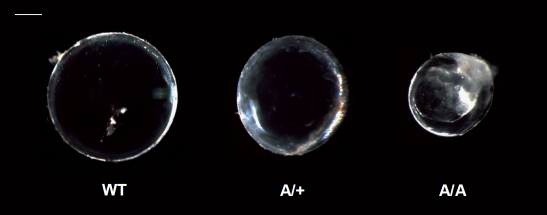
Lens opacities in *Aca47* mutants. Isolated lenses of wild-types (WT), heterozygotes (A/+), and homozygotes (A/A) are shown for 20 weeks of age. Lenses of the mutants are smaller than in the wildtype. Heterozygous lenses remain transparent, but it is obvious that homozygous lenses develop nuclear cataracts. Bar=0.5 mm.

Histologically, the eyes of ten-week-old heterozygous and homozygous mutants did not show obvious pathologic changes at the cellular level of cornea or retina (Figure 4A). The same was found for lens capsule and degradation pattern of lens fiber nuclei at the lens bow region (Figure 4B). However, further magnification of the equatorial outer cortex indicated a less regular arrangement of lens fiber layers with wave-like structures in homozygous mutant lenses. Moreover, fiber layers were partially thickened in these cases (Figure 4C). At 20 weeks of age, lenses isolated from homozygous *Aca47* mice were smaller, less stable, and exhibited cataracts in the nuclear region. Heterozygous lenses remained transparent and were less affected in size and stability, further indicating a semidominant mode of inheritance (Figure 5). Reduced eye size did not affect visual abilities as demonstrated by comparable mean spatial frequency thresholds of four-month-old heterozygotes (0.354±0.030 cyc/deg) and wild-types (0.352±0.049 cyc/deg) in the virtual vision test.

## Discussion

Here we show that a novel point mutation *Aca47* leads to smaller lenses and reduced axial eye lengths. Lenses of heterozygous individuals remain clear at least until five months of age. In contrast, homozygotes are further characterized by congenital cataracts. We identified the underlying mutation by genome-wide linkage analysis and fine mapping with microsatellite markers. It was linked to a 14.9 Mb-interval between 37.9 Mb and 52.8 Mb on chromosome 7. This interval included *Lim2* as the most likely candidate gene. The p.C51R mutation in *Lim2* is not present in other mouse strains, but co-segregates with the pathological phenotype in the *Aca47* line. Furthermore, SIFT analysis proved an amino acid exchange at this position as deleterious. Therefore, the p.C51R mutation is most likely causative for the observed pathologic eye phenotype of the *Aca47* mutant line. The cysteine residue replaced by the *Aca47* missense mutation is highly conserved among the claudin superfamily. It belongs to the signature motif of claudins (W-GWL-C-C) within the first extracellular loop. As demonstrated by the analysis with the DiANNA web tool, the two cysteines most likely form a disulfide bond.

*Aca47* is the second murine *Lim2* missense mutation described so far. A p.G15V substitution has recently been reported to induce comparable cataractogenesis and microphthalmia [15]. However, pathologic effects on lens histology were more severe than in our case including lens vacuolization and ruptured lens capsules. It was speculated that the p.G15V substitution within the first transmembrane spanning region prevents regular insertion of the protein into fiber cell membranes [15]. The semidominant nature of the p.C51R exchange in *Aca47* fits to the situation in the p.G15V mutant, which was shown to be consistent with a deleterious gain-of-function mechanism [26]. In contrast, loss-of-function mechanisms in *Lim2*-deficient mice induced only faint central cataracts in a recessive manner [16]. Regarding these facts, Lim2^C51R^ seems to be characterized by a partial rather than complete loss of function. The amino acid exchange putatively inhibits regular protein folding by preventing disulfide bond formation between Cys46 and Cys51, which might affect oligomerization processes rather than cell membrane insertion. Obviously, pathologic effects weaken fiber cell connections as indicated by reduced stability and less organized fiber layer arrangement in homozygous lenses.

Lenses of mice completely lacking *Lim2* were further characterized by refractive defects [16]. This was excluded at least for heterozygous *Aca47* mutants that responded regularly in the virtual vision tests, additionally indicating different pathologic mechanisms. Homozygous individuals completely lacked a response to the moving stripe pattern. However, this might be explained by generally observed congenital cataract formation rather than refractive errors. The regular response of *Aca47* heterozygotes indicates that slightly reduced axial eye length and lens thickness does not influence visual properties. This is in accordance to observations with the previously described Cryba2^S47P^ lens size mutant *Aca30* [27].

Reports about human *LIM2* mutations are rare. So far, p.F105V and p.G154E missense mutations have been described [28,29]. Both are associated with recessive cataracts but differ in the onset of lens opacification. Individuals affected by the p.F105V exchange develop age-related cataracts with an onset between 20 and 51 years of age [28]. In contrast, *LIM2^G154E^* induces congenital cataracts [29] according to the situation in the mouse model. The findings of this study support the possibility that additional cases of human congenital cataracts might be initiated by yet unknown *LIM2* mutations.

Finally, *Lim2* expression outside the eye is limited to very few tissues. *Lim2* transcripts were identified in blastocyst [30] and interparietal bone primordium [31] indicating a putative role of Lim2 in early embryogenesis. Further regarding data from the Allen Brain Atlas, *Lim2* appears to be weakly expressed in hippocampus and amygdalae. Consequently, additional pathologic effects of *Lim2* mutations on skeletogenesis and/or behavior remain to be excluded.

In conclusion, we described here the novel mouse mutant *Aca47*. *Aca47* carries a missense mutation in the *Lim2* gene (c.T151C; p.C51R) that leads to decreased sizes of eye axis and lens. Since homozygous mutants further develop congenital lens opacities, *Aca47* might be used as a model for congenital cataract formation in humans.
